# Nighttime Image Stitching Method Based on Guided Filtering Enhancement

**DOI:** 10.3390/e24091267

**Published:** 2022-09-09

**Authors:** Mengying Yan, Danyang Qin, Gengxin Zhang, Ping Zheng, Jianan Bai, Lin Ma

**Affiliations:** 1 Department of Electronic Engineering, Heilongjiang University, Harbin 150080, China; 2 Department of Electronics and Information Engineering, Harbin Institute of Technology, Harbin 150080, China

**Keywords:** image stitching, guided filtering, nighttime image enhancement, feature matching

## Abstract

Image stitching refers to stitching two or more images with overlapping areas through feature points matching to generate a panoramic image, which plays an important role in geological survey, military reconnaissance, and other fields. At present, the existing image stitching technologies mostly adopt images with good lighting conditions, but the lack of feature points in scenes with weak light such as morning or night will affect the image stitching effect, making it difficult to meet the needs of practical applications. When there exist concentrated areas of brightness such as lights and large dark areas in the nighttime image, it will further cause the loss of image details making the feature point matching unavailable. The obtained perspective transformation matrix cannot reflect the mapping relationship of the entire image, resulting in poor splicing effect, and it is difficult to meet the actual application requirements. Therefore, an adaptive image enhancement algorithm is proposed based on guided filtering to preprocess the nighttime image, and use the enhanced image for feature registration. The experimental results show that the image obtained by preprocessing the nighttime image with the proposed enhancement algorithm has better detail performance and color restoration, and greatly improves the image quality. By performing feature registration on the enhanced image, the number of matching logarithms of the image increases, so as to achieve high accuracy for images stitching.

## 1. Introduction

The panorama image is a seamless wide-view image generated by stitching multiple narrow-view images with overlapping areas in the same scene using image stitching technology [1]. When stitching an image, one of the source images is selected as a reference image, the other adjacent images are transformed to match the coordinate system of the reference image, and the transformation matrix is used to calculate the single response between the adjacent images to construct a panoramic image. In recent years, image stitching has become an active research area in the field of image processing and plays an important role in several applications of computer vision and computer graphics, and has been widely used in various applications, such as image rendering, medical imaging, image stabilization, 2D and 3D image mapping, satellite imaging [2], soil water balance assessment [3], and disaster prevention and control [4]. Moreover, image stitching provides support for unmanned aerial vehicle (UAV) hyperspectral remote sensing technology [5].

**Figure 1:**
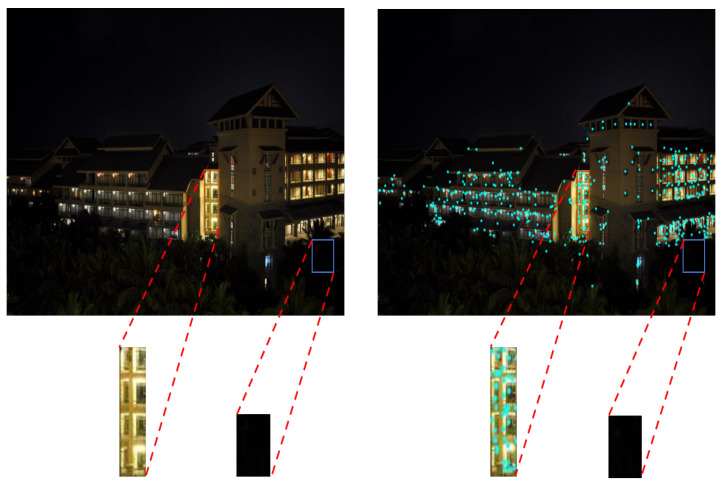
Non-uniform illumination image at night and its feature extraction image.

Most of the current mature image stitching techniques are based on clear, easy-to-process images taken in scenes with good lighting conditions, while image stitching techniques in scenes with uneven lighting, such as morning and evening, are not yet perfect. High-quality images are the basis for stitching. Due to the limitations of image capture equipment and the current capture environment, high or low illumination of the captured images can cause serious image degradation. For example, the captured nighttime images often have low signal-to-noise ratio, low brightness and low contrast. As shown in Figure 1, due to the influence of street lights or building lights, the captured nighttime images are not evenly divided, and the image brightness is relatively concentrated, while the brightness of the surrounding scene is often very dark, making it difficult to observe the dark information of the images, which makes the loss of image details a serious issue [6]. When feature extraction is performed on the image, the feature points are not extracted enough, and when stitching the night image, it is very easy to cause an image stitching failure. In addition, the night image affects the visual effect due to poor visibility, weak recognition function, and serious detail loss, resulting in the stitched image not meeting the actual application requirements. In order to improve the image quality and stitching success rate, this paper uses image enhancement techniques to preprocess nighttime images. The main contributions are summarized as follows:

- An enhancement algorithm based on guided filtering is proposed, so as to obtain nighttime images with good enhancement effect.
- A nighttime image stitching method based on enhancement algorithm is constructed to increase the number of night image matching pairs, so as to achieve high accuracy for images stitching.

## 2. Related Work

The low-illumination image enhancement algorithm mainly achieves the purpose of improving the overall contrast and brightness of the image by increasing the brightness of the dark part and suppressing the gray value of the over-bright area. As a classic problem in the field of digital image processing, the low illumination image enhancement algorithm has been developing continuously for a long time. The commonly enhancement methods of low illumination color image consist of retinex theory, gray-scale transformation, etc.

Retinex theory is a classic low-light image enhancement method. Multi-scale retinex (MSR) [7] and multi-scale retinex with color restoration (MSRCR) [8] are representative Retinex algorithms. However, these algorithms are prone to problems such as color distortion, halo, and over-enhancement. Aiming at the problem of blurred image details under low-light conditions, Liu et al. [9] proposed a low-illumination image enhancement algorithm that combines homomorphic filtering and Retinex. In RGB color space, the original image is processed using the wavelet transform and an improved Butterworth filter to obtain a detail-enhanced image. After that, in the HSV space of the original image, a color-enhanced image is obtained by using the improved bilateral filter function to process the V channel; by weighted fusion of detail-enhanced image and color-enhanced image, a high-quality image is obtained. Tang et al. [10] proposed a light map estimation method based on Retinex theory. First, the initial light map was estimated by calculating the maximum value in the three channels of R, G, and B, and anisotropic filtering was used to refine the initial light map. The illumination map is processed by adapting the gamma function, and finally, the reflection image is calculated according to the Retinex model, and the reflection image is de-sharp-masked to enhance the details.

The gamma correction function is a commonly used method in gray level transformation. The implementation method is simple, but it is usually necessary to manually set the parameters according to the characteristics of the low illumination image, and the image cannot be adaptively enhanced. Al-Ameen [11] proposed a new illumination enhancement algorithm, which employs specialized logarithmic and exponential functions to process images, and fuses the images processed by two different functions through the adaptive logarithmic processing (LIP) method. A modified S-curve function is used to improve the overall brightness of the image. Finally, low-light image enhancement is achieved by processing the image using a linear scaling function to redistribute the intensity of the image to standard dynamic range. However, the algorithm must manually set the threshold, and it is difficult to set an optimal parameter for enhancement for different scenarios.

In recent years, intelligent algorithms have developed rapidly and have also been applied to image enhancement. Qian et al. [12] proposed an adaptive image enhancement algorithm based on visual saliency, and introduced the cuckoo search algorithm and bilateral gamma adjustment function in the Hue Saturation Intensity (HSI) color space. This method improves the overall brightness of the image by finding the best parameter values for different scenes. In addition, a brightness-preserving bi-histogram construction method based on the visual saliency method (BBHCVS) is proposed to enhance the contrast of the region of interest while maintaining the image brightness. Finally, the image is adjusted using the improved saturation stretch function, which enriches the color information of the image. Considering to the characteristics of low-illumination color images, Li et al. [13] used the proposed adaptive particle swarm optimization algorithm combined with gamma correction to improve the overall brightness of the image. Furthermore, in order to enhance the saturation of the image, the image is processed using an adaptive stretching function. This method can not only improve the contrast of low illumination color images and avoid color distortion, but also effectively improve the brightness of the image and provide more detail enhancement while maintaining the naturalness of the image. Processing low-light images through intelligent algorithms improves the quality of the images. However, the introduction of intelligent algorithms undoubtedly increases the complexity of the enhancement algorithm. Moreover, image filtering algorithms are also used in image enhancement. Shan et al. [14] proposed a globally optimized linear windowed (GOLW) tone mapping algorithm, which introduces a novel highly dynamic range compression method by using local linear filtering. This algorithm realizes the enhancement of high-dynamic range (HDR) images. Noise in low-light images cannot be ignored. Hamza and Krim [15] proposed a variational approach to maximize the a posteriori estimation for image denoising, which can improve the filtering performance of Gaussian noise. Ben Hamza et al. [16] presented a variational approach to maximize the a posteriori (MAP) estimation. The approach uses geometric insight to help construct regularization functions that yield well-denoised images.

**Figure 2:**
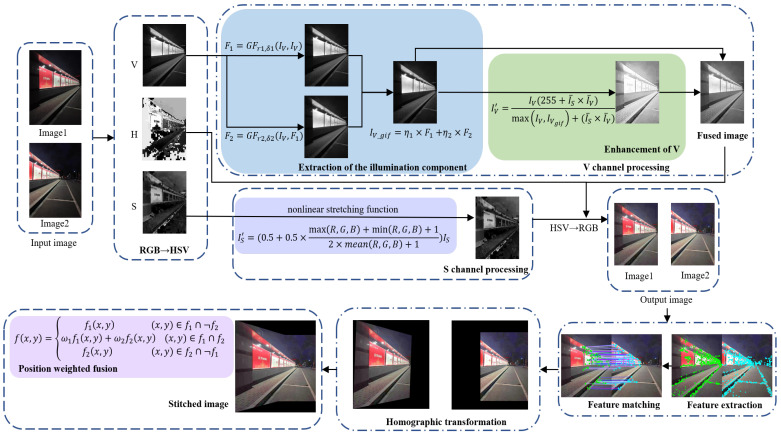
Overall framework of proposed enhancement method.

These algorithms are commonly validated using images from publicly available datasets, and are not validated for actual captured low-light images. Since there is a large amount of noise in the dark region of the actual captured nighttime images, the enhancement algorithm is very likely to amplify the noise in the dark region while enhancing the image brightness, which will have an impact on the subsequent stitching. In addition, this paper uses enhancement techniques to preprocess images, which are applied in image stitching, and if the complexity of the enhancement algorithm is too high, the stitching speed of the images will be affected. Therefore, an adaptive image enhancement algorithm based on guided filtering is proposed. First, the V component is extracted by converting the color space, and then, the illumination component is estimated by multi-scale guided filtering. The illumination components are corrected by an improved enhancement function based on the Weber–Fechner law, and an adaptive factor is introduced. The illumination components before and after the correction are combined by fusion technology, and finally transferred to the RGB color space. This algorithm achieves fast adaptive nighttime image enhancement and obtains higher quality and more detailed nighttime images, which is beneficial to the subsequent image stitching. The algorithm framework of this paper is shown in Figure 2.

The remaining contents of this paper are arranged as follows. Section 3 presents the proposed enhancement algorithm. Section 4 presents the stitching method based on the proposed enhancement algorithm preprocessing. Section 5 contains experimental results and discussions. Finally, Section 6 presents the conclusions.

## 3. The Proposed Nighttime Image Enhancement Method

### 3.1. Space Conversion

The enhancement processing on the RGB color space is easy to cause the color distortion of the image, so this paper chooses the HSV color space that is closer to the human visual expectation to enhance the image. The RGB space of the image is converted into the HSV space [17], and three components are obtained, which are *H* (hue), *S* (saturation), and *V* (luminance). The mathematical expressions are as follows:

(1) $$V=Y_{\max}$$

(2) $$S=\left\{\begin{array}{ccc} 0, & & Y_{\max}=0\\ \frac{Y_{\max}-Y_{\min}}{Y_{\max}}=1-\frac{Y_{\min}}{Y_{\max}}, & & \mathrm{otherwise} \end{array}\right.$$

(3) $$H=\left\{\begin{array}{ccc} H^{\prime }, & \; & H^{\prime }\geq 0\\ H^{\prime }+360, & \; & \mathrm{otherwise} \end{array}\right.$$

(4) $$H^{\prime }=\left\{\begin{array}{cc} 60\times \frac{G-B}{Y_{\max}-Y_{\min}}, & V=R\\ 60\times \left(\frac{B-R}{Y_{\max}-Y_{\min}}+2\right), & V=G\\ 60\times \left(\frac{R-G}{Y_{\max}-Y_{\min}}+4\right), & V=B \end{array}\right.$$

where $Y_{\max}=\max\left(R,G,B\right)$, $Y_{\min}=\min\left(R,G,B\right)$. $H^{\prime }$ can be represented by Equation (4). Through spatial transformation, the *H*, *S*, and *V* components of the image are obtained, which are expressed as $I_{H}\left(x,y\right)$, $I_{S}\left(x,y\right)$, and $I_{V}\left(x,y\right)$, respectively.

### 3.2. Estimation of Illumination Components Based on Guided Filtering

In Retinex-based image enhancement algorithms, Gaussian filtering and bilateral filtering are usually used as surround functions to estimate the light components [18]. Gaussian filtering can extract the illumination components, but the computational complexity increases significantly with the increase of the filtering window. The time complexity of the bilateral filtering is $O(Nr^{2})$, where r is the filter window radius and N is the total number of pixels in the image. When the window radius r is large or processing large-resolution images, the calculation time is too long, so the bilateral filtering method is less efficient. In addition, when a color image is smoothed by bilateral filtering, gradient inversion occurs near the edges of objects in the image, resulting in halo, which affects the quality of the output image and interferes with subsequent image processing [19].

In this paper, a linear guided filter with smoothing and edge-preserving functions is used to estimate the illuminance components. Guided filtering refers to the idea of least squares and performs operations through box filtering and integral image techniques. The time complexity is only O(N), and the execution speed is independent of the filter window size. Compared with bilateral filtering and Gaussian filtering, it is more efficient to estimate the illumination component.

Guided filtering [20] represents the output image q as a linear model related to the guide image I, the formula is as follows:

(5) $$q_{j}=a_{k}I_{j}+b_{k},\;\forall j\in \omega_{k}$$

where $q_{j}$ is the linearly transformed gray value of image *I* at pixel *j* in the window $\omega_{k}$. *k* is the center pixel of the window $\omega_{k}$. $a_{k}$ and $b_{k}$ are the linear coefficients of the guide image within a window $\omega_{k}$ of radius r centered on pixel *k*. The cost function is set as follows:

(6) $$\begin{array}{cc} E\left(a_{k},b_{k}\right) & =\sum_{j\in \omega_{k}}\left({\left(a_{k}I_{j}+b_{k}-g_{j}\right)}^{2}+\delta a_{k}^{2}\right) \end{array}$$

where δ is a regularization parameter to prevent $a_{k}$ from being too large and is used to adjust the filtering effect of the filter. The local linear coefficients $a_{k}$ and $b_{k}$ can be solved by the least square method:

(7) $$\begin{array}{cc} a_{k} & =\frac{\frac{1}{N_{\omega_{k}}}\sum_{j\in \omega_{k}}I_{i}g_{i}-\mu_{k}{\bar{g}}_{k}}{\sigma_{k}^{2}+\delta } \end{array}$$

(8) $$\begin{array}{cc} b_{k} & ={\bar{g}}_{k}-a_{k}\mu_{k} \end{array}$$

where $\mu_{k}$ and $\sigma_{k}$ are the mean and standard deviation of pixels in the window $\omega_{k}$ with radius r and center pixel *k*, respectively. ${\bar{g}}_{k}$ is the mean value of the image to be filtered in the window $\omega_{k}$. $N_{\omega_{k}}$ is the total number of pixels in the window $\omega_{k}$.

When calculating the linear coefficients of each window, it is considered that a pixel *i* can be covered by $N_{\omega_{k}}$ windows at the same time, that is, each pixel is described by multiple linear functions. Therefore, when solving the output of a certain point, it is necessary to average all the linear function values including this point, and finally get:

(9) $$\begin{array}{cc} q_{j} & =\frac{1}{N_{\omega_{k}}}\sum_{j\in \omega_{k}}\left(a_{k}I_{j}+b_{k}\right)\\ & ={\bar{a}}_{j}I_{j}+{\bar{b}}_{j} \end{array}$$

We calculate the gradient of both sides of Equation (5) simultaneously to obtain $\nabla q=a_{k}\nabla I$. It can be found that the guided filtering model has the edge preservation characteristics, and the coefficient $a_{k}$ determines the gradient preservation degree of the final image, which represents the image edge preservation degree. When $a_{k}$ is equal to 1, the output and input images have the same gradient change. When $a_{k}$ is smaller, the gradient information in $q_{j}$ is less, the smoothing force is greater, and the edge of the image is blurred. The δ in Equation (6) is a fixed regularization parameter that prevents $a_{k}$ from being too large and takes a value between 0 and 1. The smaller δ is, the smaller the smoothing multiplier of the superposition. Therefore, guided filtering uses $a_{k}$ and δ together to determine the degree of edge retention and smoothing of the output image [20,21]. Guided filtering adopts a linear method to realize the filtering process, which ensures that the output image has the gradient structure similar to the input image, and finally achieves the edge-preserving effect.

**Figure 3:**
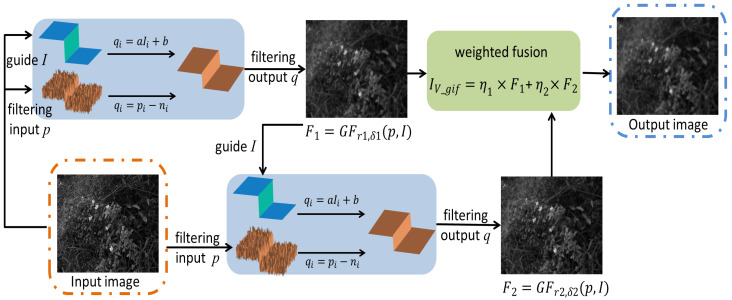
A framework for estimating illumination components based on guided filtering.

The framework of the estimation of illumination components based on guided filtering is shown in Figure 3. In this paper, we use the luminance component $I_{V}\left(x,y\right)$ as the input image and guide image. Considering the slow change of illumination in most areas, and the sudden change of brightness in local areas due to factors such as lighting, two guided filtering processes are performed on the brightness components, which are fused together by weighting as the final illumination component estimation.

(10) $$\begin{array}{cc} F_{1}\left(x,y\right) & =GF_{r1,\delta 1}\left[I_{V}\left(x,y\right),I_{V}\left(x,y\right)\right] \end{array}$$

(11) $$\begin{array}{cc} F_{2}\left(x,y\right) & =GF_{r2,\delta 2}\left[I_{V}\left(x,y\right),F_{1}\left(x,y\right)\right] \end{array}$$

(12) $$\begin{array}{c} I_{V_{-}gif}=\eta_{1}\times F_{1}\left(x,y\right)+\eta_{2}\times F_{2}\left(x,y\right) \end{array}$$

where $GF_{\left(r,\delta \right)}$ represents the guided filter function with the window radius as r and the regularization parameter δ. The weighting coefficients are $\eta_{1}=\frac{r1}{r1+r2}$, $\eta_{2}=\frac{r2}{r1+r2}$, $I_{V_{-}gif}$ denotes the filtered illumination component.

**Figure 4:**
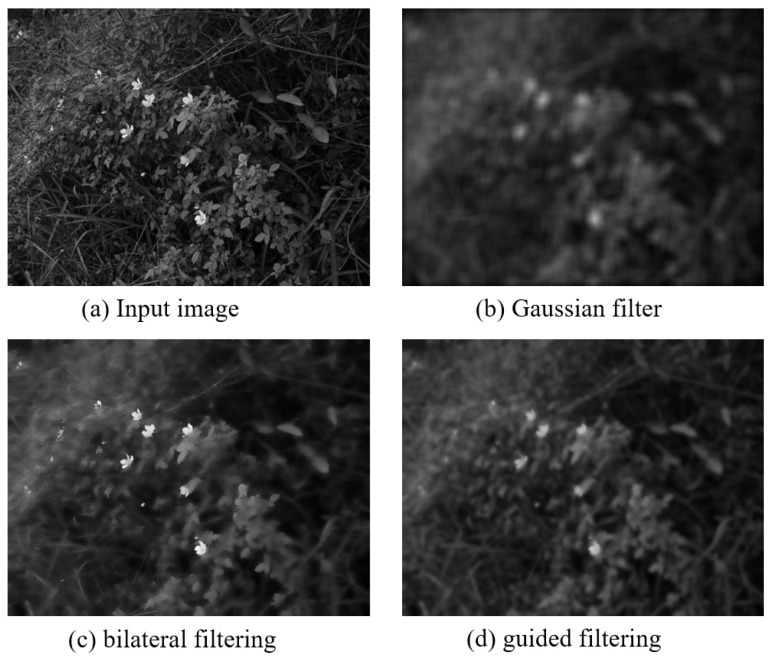
Comparison of filtering methods.

After two guided filtering processes, the illumination component image is obtained. The processed illumination component removes texture details and retains edge information, and the effect is better than Gaussian filtering and bilateral filtering. The comparison results are shown in Figure 4.

### 3.3. Adaptive Brightness Enhancement

The human eye is able to distinguish between different objects because different objects reflect light with different intensities, thus creating a contrast in brightness and color between them. The Weber–Fechner law indicates the law of the relationship between mental and physical quantities, which expresses the laws of the human visual system for the perception of the intensity of light.

Weber–Fechner’s law shows that the difference between the same visual stimulus must reach a certain ratio before it can be distinguished by the human eye, and this ratio is called the discrimination threshold of the human eye. When the brightness change is less than the discrimination threshold, the human eye cannot detect it. The threshold is not fixed, it varies with the brightness of the object’s background. Its mathematical relationship is:

(13) $$\begin{array}{cc} \Delta S & =\frac{\Delta V}{V} \end{array}$$

After integrating Equation (13), the subjective visual luminance of the human eye is obtained as

(14) $$\begin{array}{c} S\;=\;k\times \log V\;+\;c \end{array}$$

where *S* is the perceptual quantity. *k* is a constant. *V* is the physical brightness. *c* is the integral constant.

From Equation (14), it can be seen that there is a logarithmic relationship between the subjective perception of the intensity of light by the human visual system and the intensity of the stimulus change of light.

The Weber–Fechner law proves that the human visual system is a nonlinear processing process. By setting the enhancement function according to Weber–Fechner’s law, the obtained image is more in line with human vision. Due to the high complexity of logarithmic operations, the literature [22] proposed to simplify Equation (14) with Equation (15) for fitting the illumination component.

(15) $$\begin{array}{cc} I_{V}^{\prime } & =\frac{I_{V}\left(255+k\right)}{I_{V}+k} \end{array}$$

where $I_{V}^{\prime }$ is the enhanced image, $I_{V}$ is the image before enhancement, the value 255 is the gray level of the image. *k* is the adjustment coefficient. The adjustment amplitude decreases as k increases. The literature [22] adjusts the magnitude of k by the product of a weight coefficient α and the mean value of the S component. The weight coefficient α is set empirically, and the enhancement amplitude of the image is adjusted by setting different values of α. Obviously, this method cannot achieve adaptive enhancement, and the effect of the enhanced images obtained for different types of low-light image processing varies significantly.

To address this problem, this paper introduces ${\bar{I}}_{V}$ as an adaptive enhancement factor. The magnitude of enhancement is determined based on the average brightness of the image. When the brightness of the image is low, the brightness adjustment intensity of the enhancement function to the image is increased, and when the brightness of the image light is high, the enhancement intensity of the image is automatically weakened to prevent the image from being over-enhanced.

In this paper, the average luminance value is introduced as the adaptive factor of the enhancement function to realize the adaptive enhancement of the image. The adaptive enhancement function formula used is as follows:

(16) $$\begin{array}{cc} I_{V}^{\prime } & =\frac{I_{V}\left(255+{\bar{I}}_{S}\times {\bar{I}}_{V}\right)}{\max\left(I_{V},I_{V_{-}gif}\right)+\left({\bar{I}}_{S}\times {\bar{I}}_{V}\right)} \end{array}$$

where ${\bar{I}}_{S}=\frac{1}{N}\sum_{i=1}^{N}I_{S}$, ${\bar{I}}_{V}=\frac{1}{N}\sum_{i=1}^{N}I_{V}$, N is the number of pixels of image $I_{V}$.

### 3.4. Image Fusion

The image fusion technique enables the extraction of effective information from the image. In this paper, the enhanced brightness image is fused by weighted fusion and the maximum value method. The maximum value method performs fusion by comparing the size of the pixel values of the corresponding points in the image.

The maximum pixel method is used to further enhance the image when the average brightness of the input image is too low. Conversely, the average weighting method is used to prevent over-enhancement. Therefore, it is reasonable to use the average brightness value as the threshold to determine the fusion algorithm. Experiments verify that a threshold of 0.2 can achieve better enhancement effects for nighttime images.

(17) $$\begin{array}{cc} I_{V_{-}F}\left(x,y\right)= & \left\{\begin{array}{c} \max\left(I_{V_{-}gif}\left(x,y\right),I_{V}^{\prime }\left(x,y\right)\right),\;{\bar{I}}_{V}\leq 0.2\\ 0.5\times I_{V_{-}gif}\left(x,y\right)+0.5\times I_{V}^{\prime }\left(x,y\right),\;\;\mathrm{otherwise}\; \end{array}\right. \end{array}$$

where $I_{V_{-}F}\left(x,y\right)$ represents the fused image, $I_{V}$ and $I_{V}^{\prime }$ denote the images to be fused.

### 3.5. Saturation Enhancement

After the brightness of the image is increased, the saturation of the image will be reduced to a certain extent. In order to prevent the increase of brightness from affecting the saturation, an adaptive nonlinear stretching function is constructed in the literature [12] to stretch the saturation of the image. The coefficient value of the function is too small, which often leads to unsaturation when enhancing low-light images, so as to obtain images with poor visual effects. Experiments show that the supersaturation phenomenon of the image will appear as the coefficient value increases. Therefore, an improved adaptive nonlinear stretching function is proposed to enrich the image details.

The improved stretch function used in this paper is as follows:

(18) $$\begin{array}{cc} I_{S}^{\prime } & =\left(0.5+0.5\times \frac{\max(R,G,B)+\min(R,G,B)+1}{2\times \;\mathrm{mean}\;(R,G,B)+1}\right)I_{S} \end{array}$$

where $I_{S}$ and $I_{S}^{\prime }$ denote the saturation of the image before and after stretching. max(R,G,B) indicates maximum value of pixels in *R*, *G* and *B* color channels. min(R,G,B) refers to minimum value of pixels in the three channels. mean(R,G,B) refers to the average value of pixels in the three color channels.

**Figure 5:**
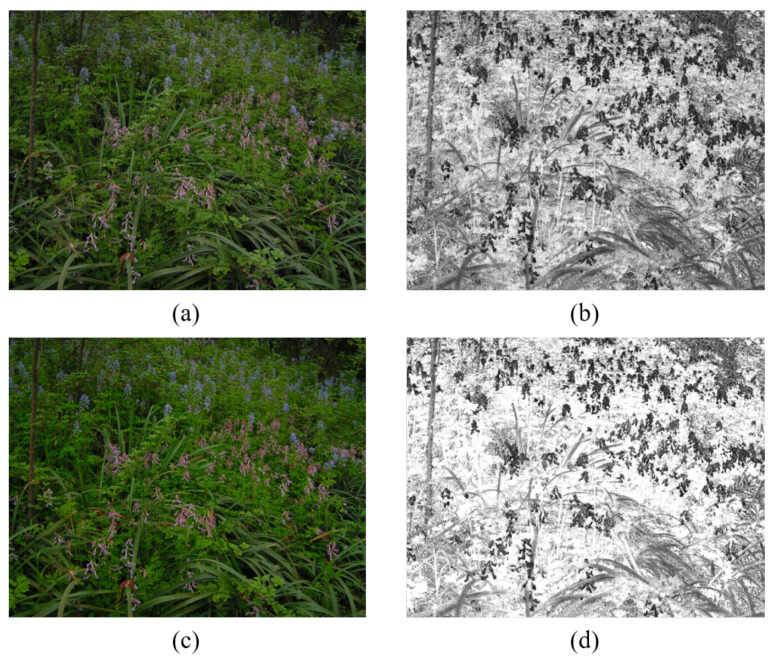
(**a**) Original image; (**b**) saturation component of original image; (**c**) nonlinear stretching result image; (**d**) saturation component of nonlinear stretching result image.

Figure 5 shows the image comparison results processed by the improved saturation stretching function. It can be seen that after stretching the S component, the image has higher saturation, and the color information of the image is more abundant.

## 4. Image Stitching Based on the Proposed Enhancement Algorithm Preprocessing

The main steps of image stitching include image preprocessing, image registration, and image fusion. After the nighttime image is preprocessed by the enhancement algorithm, the SIFT algorithm is used to extract the features, and the RANSAC algorithm is used to eliminate the mismatched pairs, and then, the transformation matrix is solved to obtain the transformation relationship between the images. Finally, the weighted position fusion algorithm is used to fuse the pixels of the spliced images to eliminate the splicing traces and generate a panoramic image.

### 4.1. Elimination of Mismatch Points by Ransac Algorithm

Considering the large number of mismatched pairs in the rough matching obtained by the SIFT algorithm, this paper uses the RANSAC (Random Sampling Consensus) algorithm to eliminate the mismatched pairs. The RANSAC algorithm regards the data that meet the estimated model as an interior point, and the data that do not conform to the estimated model as an exterior point. Through parameter estimation, a reasonable result under a certain probability is generated, and repeated testing and continuous iteration increase the probability. When the number of iterations is sufficient over time, the true model is estimated from the dataset.

Assuming that the global homography matrix to be solved is H, the error threshold ε is set, and the number of iterations is k, the operation steps of the RANSAC algorithm to eliminate the mismatch points are as follows:

1. Randomly select 4 groups of non-collinear matching point pairs from the rough matching results;
2. Solve the projection transformation matrix H according the selected matched pairs of points;
3. Among the remaining matching pairs, apply the H derived from the above step to count the reprojection error less than the set threshold ε of the matching pairs, noting the matching pair as an inner point and counting the number.
4. If the number of current interior points is greater than the previous optimal projection transformation, the current projection transformation is recorded as the optimal projection transformation;
5. If the current probability is within the range allowed by the model or the number of iterations is greater than the specified number of times, the calculation is completed. If it does not meet the requirements, the above process is repeated until the requirements of the model are met or the specified number of iterations is completed.

Through the processing of RANSAC algorithm, the homography matrix of global projection transformation is obtained while eliminating the mismatched pairs, which represents the optimal spatial transformation relationship between the two images to be spliced.

### 4.2. Fusion of Stitched Images

Image fusion is the process of combining two images to be stitched together in a common coordinate system. In order to make the resulting stitched image more natural, it is necessary to fuse the overlapping parts of the two images to be stitched together.

This paper adopts the position-weighted fusion algorithm. The position-weighted fusion algorithm is a gradual and gradual-out fusion algorithm. When calculating the fusion transition area pixels, the overlapping area pixels are generated with linear weights. The formula is as follows:

(19) $$\begin{array}{cc} f(x,y) & =\left\{\begin{array}{cc} f_{1}\left(x,y\right), & \left(x,y\right)\in \left(f_{1}\cap \neg f_{2}\right)\\ \omega_{1}f_{1}\left(x,y\right)+\omega_{2}f_{2}\left(x,y\right), & \left(x,y\right)\in \left(f_{1}\cap f_{2}\right)\\ f_{2}\left(x,y\right), & \left(x,y\right)\in \left(f_{2}\cap \neg f_{1}\right) \end{array}\right. \end{array}$$

where $\omega_{1}$ and $\omega_{2}$ are the pixel weighting coefficients corresponding to the images $f_{1}$ and $f_{2}$, respectively, which control the smooth transition of the overlapping area from the left border to the right border. The calculation formula is as follows:

(20) $$\left\{\begin{array}{c} \omega_{1}=\frac{x-L}{R-L}\\ \omega_{2}=1-\omega_{1} \end{array}\right.$$

where *L* and *R* are the left and right boundaries of the overlapping region, respectively. The weight of the position-weighted fusion algorithm changes with the width of the overlapping area, so as to realize the smoothness of the pixel change in the fusion area, which can effectively improve the hard boundary effect of the stitched image, and realize the slow transition from the reference image to the target image in the overlapping part.

## 5. Experiments and Discussions

### 5.1. Experiment Setting

For the proposed image enhancement algorithm, specific images are used for validation, followed by feature matching and stitching for comparison. All experiments in this research were run on MATLAB R2018a on a PC with 1.6 GHz CPU and 8 GB RAM.

To evaluate the effectiveness of the proposed enhancement algorithm, we compare the proposed method with conventional image enhancement algorithms and state-of-the-art technologies, i.e., multi-scale retinex (MSR) [7], multi-scale retinex with color restoration (MSRCR) [8], retinex-based Multiphase algorithm (RBMP) [23], and adaptive image enhancement method (AIEM) [22]. Six representative images with uneven illumination (image #1–6) are selected from the MEF [24] and NPE [18] image sets and combined with four nighttime images actually taken as the experimental test images (image #7–10). The pictures collected in this article were taken in front of the tennis court and dormitory building of Heilongjiang University. This experiment evaluates the proposed enhancement algorithm and other comparison algorithms in terms of both subjective evaluation and objective evaluation metrics. The subjective visual evaluation of images can truly reflect the image quality from the visual perspective, and the evaluation is simple and reliable. The objective evaluation metrics judge the image quality from the specific metric level.

The relevant parameters of the algorithm are set as follows:

1. In order to balance the smoothness of the image and the edge-holding effect, this paper sets the guided filtering parameters as $r_{1}=3$, $r_{2}=5$, $\delta_{1}=0.14$, $\delta_{2}=0.14$.
2. The AIEM algorithm uses the parameters in the authors’ original paper, and the 3 Gaussian scale parameters are: $\sigma_{1}=15$, $\sigma_{2}=80$, $\sigma_{3}=250$, the weights are set as $\alpha_{1}=0.1$, $\alpha_{2}=1$.

### 5.2. Subjective Evaluation of Image Enhancement

**Figure 6:**
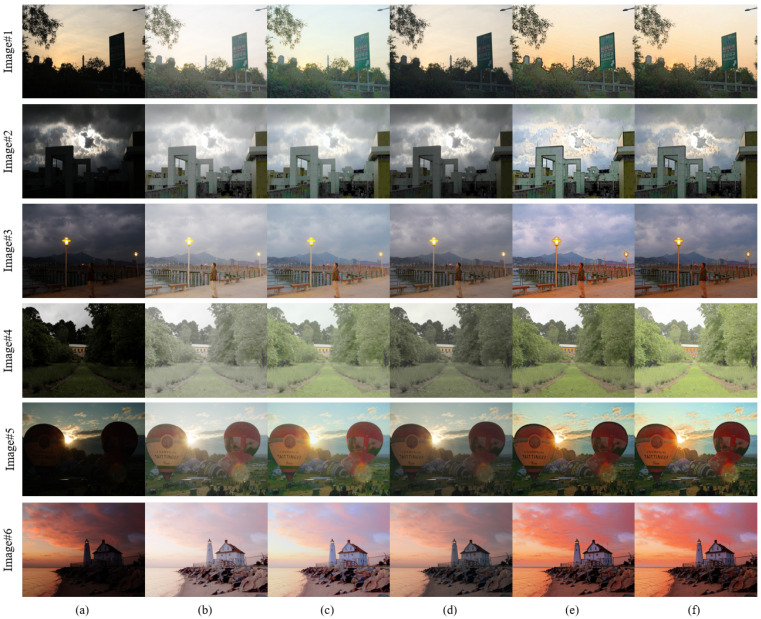
Comparison with various methods on the dataset image. (**a**) Original images. (**b**) MSR results. (**c**) MSRCR results. (**d**) RBMP results. (**e**) AIEM results. (**f**) Our results.

The unevenly illuminated images in the public low-light dataset are processed using different enhancement algorithms, and the results are shown in Figure 6. The brightness of the image processed by the MSR algorithm is improved, but there is an over-enhancement phenomenon, and the overall image appears white, such as the clouds in image #2 (b) and yellow houses in image #4 (b). Image details are lost due to excessive image brightness enhancement. The MSRCR algorithm can improve the brightness of the image, but the color preservation effect of the image is still poor. For example, the sky color of image #1 (c) and image #6 (c) cannot maintain the color effect in the original image. The overall color of the image is lighter, with obvious color distortion. The brightness of the dark areas of the image processed by the RBMP algorithm is not significantly improved, and the color retention ability is slightly insufficient, such as the street signs in image #1 (d) and the balloons in image #5 (d). The color preservation effect of the image processed by the AIEM algorithm is good, but the halo phenomenon occurs in the alternating light and dark areas, such as around the street lights in image #3 (e). In addition, the images processed by the AIEM algorithm have artifacts on the edges of foreground objects, which affect the visual effect of the image, such as the edges of buildings and the edges of alternating light and dark clouds in image #2 (e). The brightness of the dark area of the image processed by the algorithm proposed in this paper is improved, and there is no overexposure phenomenon, and the color preservation effect is close to that of the AIEM algorithm. Due to the introduction of guided filtering, the edge of the image processed by the proposed method is sharper, such as the edge of the house in image #4 (f) and the edge of the lighthouse in image #6 (f). The image processed by the proposed algorithm has more natural brightness processing at the intersection of light and dark, without halos and artifacts. As shown in image #1 (f), the edge of the sign is clear and the color transition is natural.

**Figure 7:**
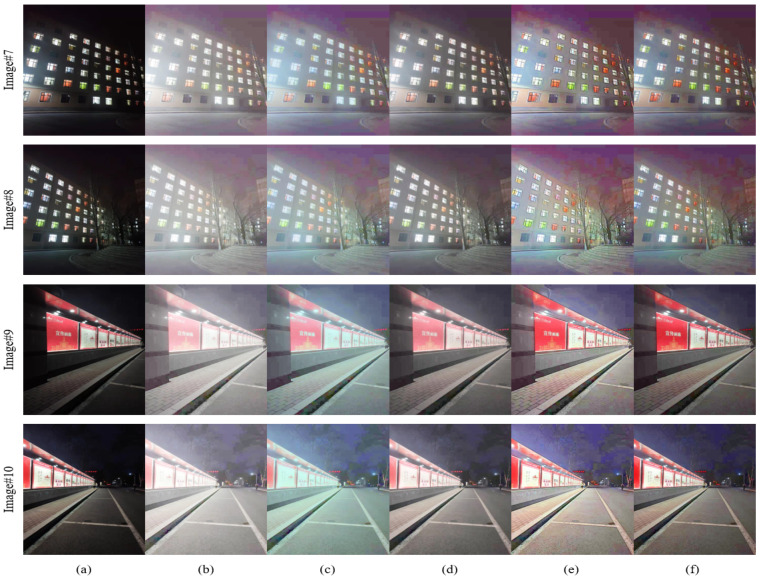
Comparison with various methods on the collecting image. (**a**) Original images. (**b**) MSR results. (**c**) MSRCR results. (**d**) RBMP results. (**e**) AIEM results. (**f**) Our results.

The collected nighttime images (images #7–10) were enhanced using different algorithms, and the results are shown in Figure 7. The MSR algorithm improves the overall brightness of the image, but also for high-brightness areas, where overexposure occurs at the light source, as shown in the brightness area of images #6–7 (b). The MSRCR algorithm also has an overexposure phenomenon, the overall picture is bluish, and the “block effect” in the dark area is obvious, which affects the visual effect of the image, such as the window areas of image #7 and image #8. Compared with the MSR and MSRCR algorithms, the enhancement effect of the RBMP algorithm is improved, and the brightness of the dark areas of the image is improved, such as the steps and trees in image #8 (d). This algorithm improves the image over-enhancement phenomenon, but the detail preservation effect in the brightness area is still not good, such as the window in image #8 (d) and the light sign area in image #9 (d), the brightness enhancement is unnatural. The AIEM algorithm has a better effect on color retention, but in the edge area where light and dark alternate, such as in image #7 (e) and image #8 (e), there are the artefacts around the window, which affect the visual effect. In addition, the color of the image processed by AIEM algorithm is unnatural, such as the color of the light sign in image #9 (e) and image #10 (e). The image processed by the proposed algorithm maintains good brightness in areas with strong illumination, and improves the brightness in dark areas. As shown in image #7 (f) and image #8 (f), the edges of the windows are sharp, and the images have moderate brightness and good color retention. The brightness and color of the lights in image #9 (f) and image #10 (f) are natural with no over-enhancement.

### 5.3. Objective Evaluation of Image Enhancement

In order to objectively reflect the enhancement effect of each algorithm in processing low-light images, this paper uses average value (AVG), average gradient (AG), information entropy (IE), and peak signal-to-noise ratio (PSNR) to measure the quality of the enhanced low-light images [25,26,27].

The mean of the image is used to represent the average brightness of the image. The calculation formula is given by Equation (21).

(21) $$\begin{array}{c} \;\mathrm{AVG}\;=\frac{\sum_{i=1}^{M}\sum_{j=1}^{N}I\left(i,j\right)}{M\times N} \end{array}$$

where *M* is the image height, *N* is the image width. I(i,j) refers to the gray value of the pixels in row *i* and column *j* of the image.

The average gradient is used to measure the sharpness of the image. The larger the average gradient of the image, the more layers of the image, and the clearer the image. *AG* is calculated by Equation (22).

(22) $$\begin{array}{c} AG=\frac{1}{M\times N}\sum_{i=1}^{M}\sum_{j=1}^{N}\sqrt{\frac{{\left(\frac{\partial f}{\partial x}\right)}^{2}+{\left(\frac{\partial f}{\partial y}\right)}^{2}}{2}} \end{array}$$

where $\left(\frac{\partial f}{\partial x}\right)$ and $\left(\frac{\partial f}{\partial y}\right)$ respectively represent the horizontal and vertical gradients of the M×N image.

Information entropy is an index used to measure the richness of image information. The greater the image information entropy, the better the detail performance of the image. The Information entropy (IE) is calculated by Equation (23).

(23) $$\begin{array}{cc} H & =-\sum_{x\in k}q\left(x\right)\ln q\left(x\right) \end{array}$$

where q(x) represents the distribution density of the image gray level *x*. *k* is the gray level of the image.

The peak signal-to-noise ratio is used to measure the degree of image distortion or the anti-noise level. The larger the value, the smaller the image distortion and the higher the anti-noise level. *PSNR* is calculated by Equation (24).

(24) $$\begin{array}{cc} PSNR & =10\log_{10}\left(\frac{{\left(\max\left(I_{i}\right)\right)}^{2}}{MSE}\right) \end{array}$$

where max$\left(I_{i}\right)$ is the maximum gray level value of the input image $I_{i}$. *MSE* is the Mean Square Error of the enhanced image and the input image. *MSE* is given by Equation (25).

(25) $$\begin{array}{cc} MSE & =\frac{1}{M\times N}\sum_{i=1}^{M}\sum_{j=1}^{N}{\left(x\left(i,j\right)-y\left(i,j\right)\right)}^{2} \end{array}$$

where x(i,j) is the gray value of the pixels in row *i* and *j* column of the orignal image. y(i,j) is the gray value of the pixels in row *i* and column *j* of the enhanced image.

Table 1 lists the comparison of various indicators of the 6 dataset images enhanced by different algorithms. It can be seen from Table 1 that the average value of the processed images is improved, indicating that the brightness of the image is enhanced, but because the MSR and MSRCR over-enhance the image, the image is white, so the average value is too large. The average value of the image enhanced by the proposed algorithm is moderate, which shows that the brightness of the image is adaptively enhanced, and there is no over-enhancement phenomenon, which is in line with the human eye observation effect. From the point of view of the average gradient, the five enhancement algorithms all improve the image clarity to a certain extent, among which the proposed algorithm and AIEM have better average gradient values. The image information entropy values processed by each enhancement algorithm are improved, among which AIEM and the proposed algorithm obtain relatively high values. It can be seen from the PSNR value that the AIEM and RBMP algorithms and the proposed algorithm have better effect on image noise suppression.

Table 2 lists the comparison of the evaluation indexes of the 4 nighttime images actually shot through 5 different enhancement algorithms. As shown in Table 2, the mean value of the images enhanced by MSR and MSRCR is still too high, indicating that the image has been enhanced and the peak signal-to-noise of the image is low. After the image is enhanced by the proposed algorithm, the mean value is increased compared with the original image, but the brightness of the enhanced image is moderate, and there will be no excessive enhancement. The image processed by the proposed algorithm has the highest PSNR value, indicating that the suppression effect of nighttime image noise is better than other algorithms. Although the IE or AG values of individual images processed by AIEM are higher than those obtained by the method proposed in this paper, the comprehensive performance of our method is much better than that of the other methods.

In general, the proposed image enhancement algorithm can effectively improve image brightness and clarity. In addition, more detailed texture information of the image can be recovered, the color information is also protected, and the noise in the dark place is suppressed, resulting in a higher quality image, which is conducive to subsequent stitching.

### 5.4. Time Complexity

Table 3 shows the processing time comparison of each algorithm. The MSRCR algorithm requires Gaussian filtering of the logarithmic domains of R, G, and B components of the original image to estimate the illumination components, so the complexity is higher; RBMP uses gamma-corrected sigmoid function processing for image enhancement, which is a simple method and less complex than the MSR algorithm. The AIME algorithm is less time consuming than MSR and MSRCR, but it employs multiscale Gaussian filtering to extract the illumination components, which leads to an increase in the running time and a sharp increase in the complexity of the algorithm as the Gaussian window increases. Compared with the AIEM algorithm, the proposed algorithm uses guide filtering in estimating the illuminance components, which reduces the complexity of the algorithm and makes the processing time decrease, and lays the foundation for the subsequent fast stitching.

### 5.5. Feature Matching

**Figure 8:**
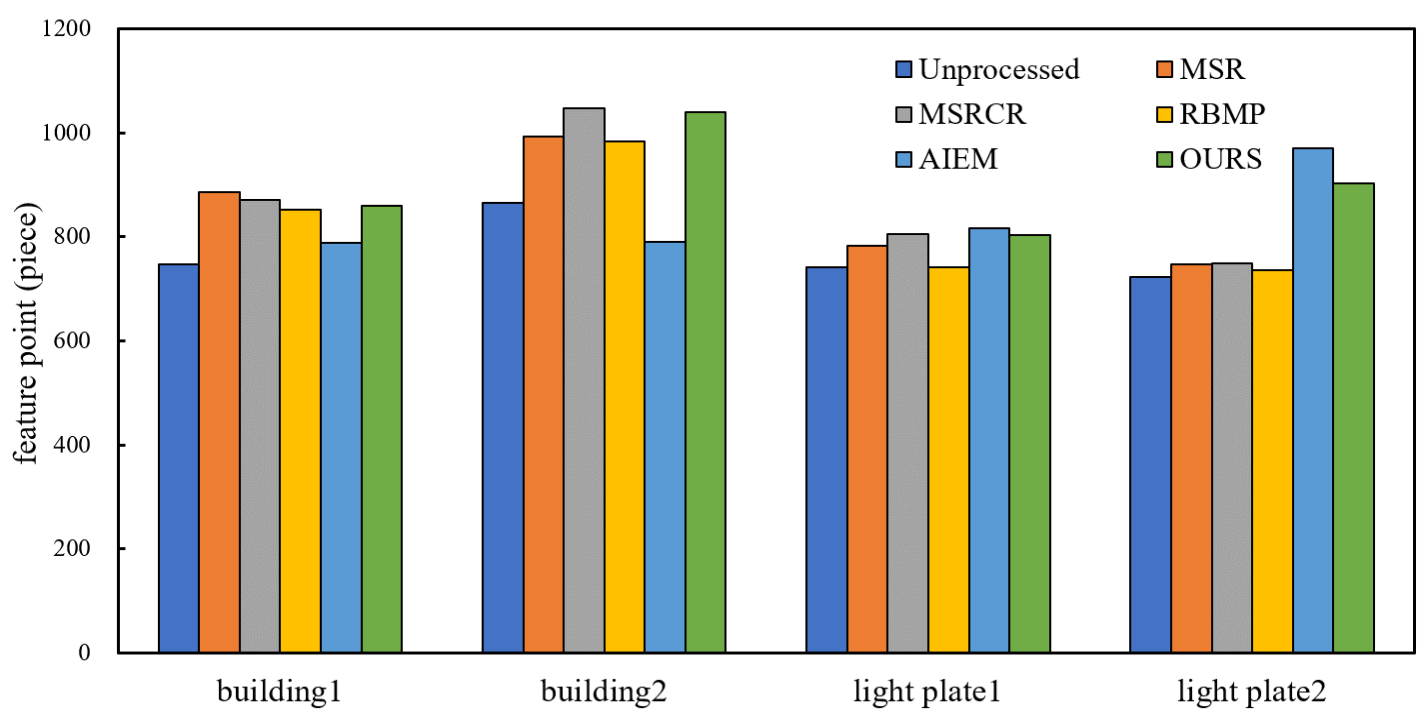
Comparison of the number of feature points.

**Figure 9:**
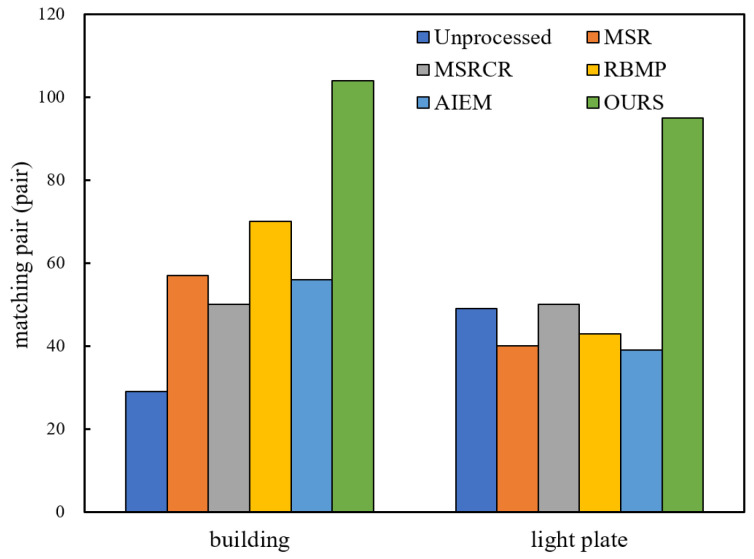
Comparison of feature point logarithms.

**Figure 10:**
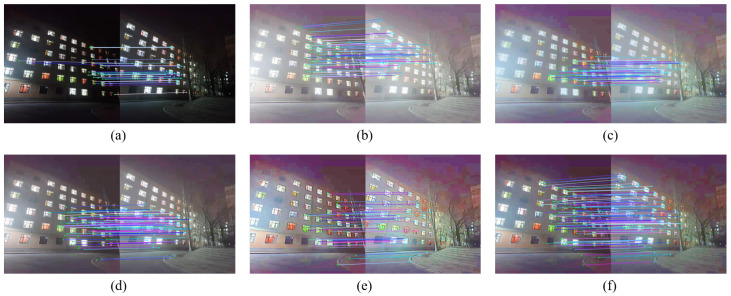
Comparison of feature matching for ‘building’ image. (**a**) Original image. (**b**) MSR result. (**c**) MSRCR result. (**d**) RBMP result. (**e**) AIEM result. (**f**) Our result.

**Figure 11:**
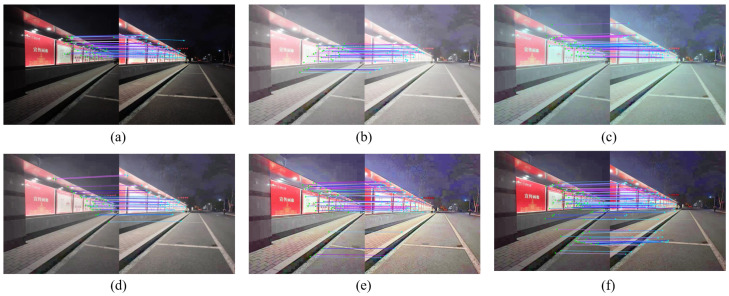
Comparison of feature matching for ‘light plate’ image. (**a**) Original image. (**b**) MSR result. (**c**) MSRCR result. (**d**) RBMP result. (**e**) AIEM result. (**f**) Our result.

For the sake of description, image #7 and #8 are named ‘building1’, ‘building2’, image #9 and #10 are named ‘light plate1’, ‘light plate2’. After enhancing the images with different enhancement algorithms, the SIFT algorithm in the VLFeat library was used for feature extraction and matching. The comparison of the number of feature points extracted and the number of matched pairs are shown in Figure 8 and Figure 9, and the matching results are shown in Figure 10 and Figure 11.

As can be seen from the data comparison in Figure 8, the number of feature points extracted from the enhanced nighttime image increases significantly, among which the feature extraction effect of the proposed algorithm is more significant for four nighttime images. The extraction ability is relatively stable, and will not fluctuate greatly due to different images. Figure 9 shows that the number of correctly matched feature pairs is greatly improved for the images enhanced by the proposed algorithm.

Figure 10a and Figure 11a show that the matched feature points of the images before enhancement are fewer and mainly concentrated in the regions with stronger lighting, while there are almost no successfully matched feature points in the dark places. When stitching the nighttime images with uneven illumination, the feature points are clustered in the bright places, which makes the obtained transformation matrix error large and eventually leads to poor stitching. As shown in Figure 10f and Figure 11f, through the proposed enhancement algorithm, the dark area of the road surface is matched to the feature points. This experiment proves that the proposed enhancement algorithm is beneficial to the feature extraction and registration of images with nighttime images, and provides guarantee for subsequent stitching.

### 5.6. Image Stitching

**Figure 12:**
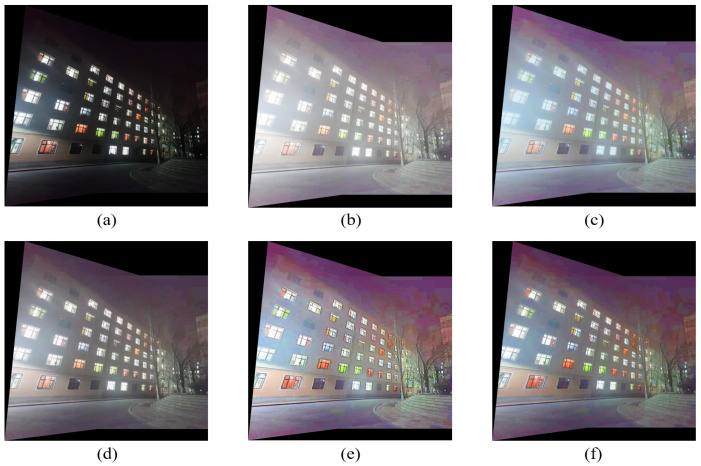
Comparison of stitching of ‘building’ image. (**a**) Original image. (**b**) MSR result. (**c**) MSRCR result. (**d**) RBMP result. (**e**) AIEM result. (**f**) Our result.

**Figure 13:**
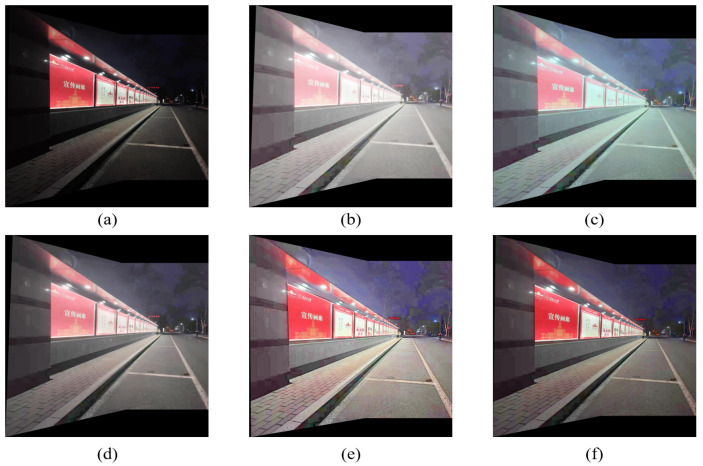
Comparison of stitching of ‘light plate’ image. (**a**) Original image. (**b**) MSR result. (**c**) MSRCR result. (**d**) RBMP result. (**e**) AIEM result. (**f**) Our result.

The two groups of images of ‘building’ and ‘light plate’ are spliced. The spliced images are shown in Figure 12 and Figure 13. The comparison of evaluation indicators is shown in Table 4.

After the image is preprocessed by the enhancement algorithm, the details of the image are more abundant, and the information of the dark area of the image is enhanced. Objects originally in dark areas, such as steps and trees in Figure 12f, can be clearly observed after enhancement. It can be observed from Figure 13a that when splicing the original image, there is an obvious ghost at the step, which is caused by the inaccuracy of the transformation matrix due to insufficient matching logarithms. After stitching using the comparison enhancement algorithm, as shown in Figure 13b–f, the ghosting phenomenon is improved, but not eliminated. It can be seen from Figure 13f that the ghosting phenomenon at the steps in the image disappears after stitching and after enhancement by the proposed algorithm, indicating that the proposed algorithm can obtain matching pairs with better quality, and then solve a more accurate transformation matrix, which improves the stitching accuracy.

As indicated in Table 4, the stitched images processed by the enhancement algorithm have improved in mean, average gradient, information entropy, and signal-to-noise ratio, indicating that the quality of the stitched image can be effectively improved by using the enhancement algorithm to preprocess the image. The MSR and MSRCR algorithms over-enhance bright areas, resulting in too large average image values and dazzling images. The five enhancement algorithms have little difference in the improvement of information entropy, indicating that the enhancement algorithms all enrich the image details. The AG value of the images processed by the proposed algorithm is slightly lower than that of the AIEM algorithm. The image processed by the proposed enhancement algorithm has the highest PSNR value, indicating that the proposed algorithm can improve the brightness while suppressing noise. Overall, the proposed algorithm improves nighttime image quality and achieves better image quality, which supports practical applications.

## 6. Conclusions

Aiming at the problem of the poor nighttime image stitching effect, an enhancement algorithm applicable to nighttime image stitching is proposed. The V component obtained by converting the color space of the image is used to extract the lighting component of the scene via multi-scale guided filtering. Then, the correction function based on the Weber–Fechner law is used to enhance the light component, and an adaptive factor is introduced to realize the adaptive brightness enhancement. Additionally, the S component is processed using a nonlinear stretching function. Finally, a nighttime image with better enhancement effect is obtained through color space conversion.

In this paper, the proposed method is verified by selected low-illumination dataset images and the collected nighttime images, and compared with four other enhancement algorithms. From the experimental results, it can be seen that the images with rich details, good color retention, high signal-to-noise ratio, and rich texture information are obtained by processing the image through the proposed enhancement algorithm. Compared with other algorithms, the proposed algorithm has the lowest complexity and can meet the demand of fast stitching. By performing feature matching on the enhanced image, more matching logarithms can be obtained. The proposed method has higher stitching accuracy. In conclusion, the proposed adaptive enhancement method based on guided filtering can meet the requirements of fast and efficient nighttime image stitching, which provides value for the application of nighttime surveillance image stitching.

## Figures and Tables

**Table 1:** Objective Evaluation Metrics for dataset Images.

Image Index	Methods	AVG	AG	IE	PSNR
Image#1	Unprocessed	106.2343	8.3369	6.9185	
	MSR	175.9547	8.7906	6.4680	10.3850
	MSRCR	168.3584	8.2684	7.0153	11.3675
	RBMP	135.2965	8.9986	6.9556	17.2985
	AIEM	147.3297	14.3700	7.5040	14.0416
	OURS	144.5374	13.2811	7.3775	14.5312
Image#2	Unprocessed	48.2641	2.0051	6.8375	
	MSR	145.1272	3.3525	6.5330	7.8575
	MSRCR	143.5546	3.3842	7.3012	8.0074
	RBMP	104.2055	2.7525	6.7742	12.4023
	AIEM	148.2635	5.5219	7.4767	7.3040
	OURS	120.7855	4.4995	7.4282	10.1089
Image#3	Unprocessed	48.2371	1.3825	6.7409	
	MSR	166.7816	2.4035	7.0180	6.6202
	MSRCR	149.7294	2.3618	7.0570	7.8877
	RBMP	119.2852	2.1157	7.2369	11.0260
	AIEM	144.3214	4.1107	7.4897	8.2008
	OURS	106.7472	2.9637	7.2551	12.5561
Image#4	Unprocessed	42.8373	2.1314	6.0142	
	MSR	155.9016	3.5015	6.7860	6.9969
	MSRCR	154.5647	3.4134	7.0101	7.3259
	RBMP	103.6729	3.5788	6.8242	11.9598
	AIEM	117.5364	5.9300	7.1468	10.0802
	OURS	152.4410	7.2409	7.2610	6.8059
Image#5	Unprocessed	41.5932	2.6622	6.0279	
	MSR	132.5138	5.4725	6.1532	8.6901
	MSRCR	128.1398	6.6046	5.7902	9.1541
	RBMP	91.5238	4.3335	7.4906	13.3060
	AIEM	95.9058	5.2659	7.4171	12.0187
	OURS	108.3902	6.0585	7.4950	10.1912
Image#6	Unprocessed	68.7553	2.9095	7.4913	
	MSR	163.0143	4.0134	7.1714	7.2353
	MSRCR	172.4398	3.9305	7.4066	7.9513
	RBMP	121.1455	3.5583	7.5343	13.2318
	AIEM	120.7220	5.2254	7.7427	12.5621
	OURS	119.3843	4.8976	7.8425	12.6054

**Table 2:** Objective evaluation index of collected images.

Image Index	Methods	AVG	AG	IE	PSNR
Image#7	Unprocessed	41.3997	2.9346	6.4685	
	MSR	150.8871	2.1873	7.0354	7.0697
	MSRCR	134.3781	2.1236	7.1479	8.2082
	RBMP	105.6925	2.6895	7.1079	11.8398
	AIEM	123.9911	4.3936	7.3721	9.3905
	OURS	102.2740	3.5814	7.1135	12.1219
Image#8	Unprocessed	41.0353	2.5788	6.3299	
	MSR	157.8570	1.9959	6.8064	6.5846
	MSRCR	143.3581	1.9618	6.9119	7.6316
	RBMP	110.1061	2.4717	6.9227	11.1967
	AIEM	135.3832	4.2874	7.2146	8.3248
	OURS	110.3024	3.2543	6.9137	11.0764
Image#9	Unprocessed	48.8969	2.8815	6.8573	
	MSR	158.1191	2.4676	7.3004	7.0982
	MSRCR	143.4791	2.5297	7.3939	8.2073
	RBMP	109.2115	2.8726	7.0313	12.3069
	AIEM	133.7210	4.9648	7.5166	8.9848
	OURS	92.9747	4.0274	7.2507	14.6012
Image#10	Unprocessed	48.8969	3.9367	7.0113	
	MSR	165.9230	3.4260	7.2678	7.0697
	MSRCR	149.2280	3.4045	7.3891	8.2082
	RBMP	117.8824	4.1512	7.3876	12.0753
	AIEM	141.2429	7.0486	7.5878	8.9907
	OURS	99.3570	5.6238	7.4180	14.8902

**Table 3:** Comparison of different methods on computational complexity.

Image Index	Size	MSR (s)	MSRCR (s)	RBMP (s)	AIEM (s)	OURS (s)
Image#1	533 × 800	0.5618	1.1672	0.7213	0.5870	0.3854
Image#2	399 × 700	0.5216	0.9731	0.5211	0.3967	0.2399
Image#3	960 × 1280	1.1909	1.7947	1.1530	0.8450	0.9827
Image#4	1728 × 2592	3.4892	5.8912	3.2968	3.8341	3.5704
Image#5	339 × 512	0.4320	0.7815	0.5012	0.3445	0.2308
Image#6	340 × 512	0.2708	0.7146	0.5434	0.3635	0.2313
Image#7	1280 × 916	1.4676	2.8420	1.3880	1.2532	0.9239
Image#8	1280 × 916	1.4965	2.8091	1.2366	1.1743	0.9160
Image#9	1280 × 916	1.4500	2.8855	1.2483	1.2714	0.9767
Image#10	1280 × 916	1.6385	2.8574	1.2577	1.2801	0.9079

**Table 4:** Objective evaluation index of collected images.

Image Index	Methods	AVG	AG	IE	PSNR
building	Unprocessed	37.0289	1.8757	5.9837	
	MSR	127.5154	1.6164	6.3534	7.7301
	MSRCR	115.3205	1.5652	6.3684	8.9447
	RBMP	90.8140	1.8731	6.4962	11.9741
	AIEM	108.2368	3.0621	6.6693	9.5082
	OURS	88.4005	2.3571	6.4294	12.3991
light plate	Unprocessed	36.1945	1.8956	6.1002	
	MSR	122.8710	1.9617	6.5805	8.0107
	MSRCR	109.6623	1.8978	6.5875	9.1976
	RBMP	83.1930	2.1151	6.6198	13.0639
	AIEM	109.0777	4.0151	7.0048	9.1821
	OURS	72.6646	2.9110	6.6502	13.9791

## Data Availability

The data presented in this study are available on request from the corresponding author. The data are not publicly available due to privacy.
